# Prevalence of gestational, placental and congenital malaria in north-west Colombia

**DOI:** 10.1186/1475-2875-12-341

**Published:** 2013-09-23

**Authors:** Olga Agudelo, Eliana Arango, Amanda Maestre, Jaime Carmona-Fonseca

**Affiliations:** 1 Grupo Salud y Comunidad, Facultad de Medicina, Universidad de Antioquia, Medellín, Colombia

**Keywords:** Pregnancy, Malaria, *Plasmodium*, Prevalence, Colombia

## Abstract

**Background:**

The frequency of pregnancy-associated malaria is increasingly being documented in American countries. In Colombia, with higher frequency of *Plasmodium vivax* over *Plasmodium falciparum* infection, recent reports confirmed gestational malaria as a serious public health problem. Thick smear examination is the gold standard to diagnose malaria in endemic settings, but in recent years, molecular diagnostic methods have contributed to elucidate the dimension of the problem of gestational malaria. The study was aimed at exploring the prevalence of gestational, placental and congenital malaria in women who delivered at the local hospitals of north-west Colombia, between June 2008 and April 2011.

**Methods:**

A group of 129 parturient women was selected to explore the prevalence of gestational, placental and congenital malaria in a descriptive, prospective and transversal (prevalence) design. Diagnosis was based on the simultaneous application of two independent diagnostic tests: microscopy of thick blood smears and a polymerase chain reaction assay (PCR).

**Results:**

The prevalence of gestational malaria (thick smear /PCR) was 9.1%/14.0%; placental malaria was 3.3%/16.5% and congenital malaria was absent. A history of gestational malaria during the current pregnancy was significantly associated with gestational malaria at delivery. *Plasmodium vivax* caused 65% of cases of gestational malaria, whereas *P. falciparum* caused most cases of placental malaria.

**Conclusions:**

Gestational and placental malaria are a serious problem in the region, but the risk of congenital malaria is low. A history of malaria during pregnancy may be a practical indicator of infection at delivery.

## Background

Women inhabiting malaria endemic areas are at variable risk of acquiring gestational malaria according to the degree of endemicity and their parity. In regions of high stable transmission, they might acquire significant clinical immunity before pregnancy, and placental malaria is often asymptomatic, but it can produce severe maternal anaemia and foetal growth restriction [1]. In such regions, placental malaria is common in first-time mothers, and women develop specific immunity against placental infection over successive pregnancies. On the other hand, in areas of low unstable transmission, malaria is often symptomatic, regardless of parity, and it might result in foetal loss and maternal death [2].

In the Americas, the problem of gestational malaria has been the subject of few reports [3-6]. The frequency of gestational malaria and placental malaria in the region of Uraba, north-west Colombia, is relatively high, affecting 10–12 out of 100 pregnant women, when measured by microscopy [7]. A report based on samples from the same region (2004–2006) found a prevalence of gestational malaria of 14.0% and placental malaria of 10.5% (by thick smear microscopy) [8]. Another study on *Plasmodium* infection at the time of delivery, reported a prevalence of gestational malaria of 13% with microscopy, but the frequency increased to 32% with a polymerase chain reaction (PCR) assay [9]. In addition, the histopathological examination of placental tissue showed unmistakable signs of placental malaria in 19% of the 79 specimens studied, in which 33% were characterized as acute, 7% as chronic and 60% as past infections [9]. The diagnostic capacity of histopathology was superior to that of microscopy and PCR, mainly because the basis of the histopathology diagnosis is the observation of the parasite and/or the malarial pigment.

Recent evidence gathered in the country, confirmed the deleterious effect of the infection, in both *P. vivax* and *P. falciparum* malaria, in pregnant subjects, with presence of organ specific complications [10], congenital malaria [11], and low birth weight and prematurity [12]. All the above data confirmed gestational and placental malaria as a serious public health problem in north-west Colombia, where most of the country´s cases of malaria are reported. In addition, these reports confirmed that both *P. falciparum* and *P. vivax* are the causative species in these conditions, with 65-70% of reported cases due to *P. vivax*[13,14].

Resistance of *P. falciparum* to anti-malarials is widespread in the country [15,16], and artemisinin-based combination therapy is recommended from 2006, with two different schemes applied between 2006–2009: a three-day course of artesunate plus mefloquine for the Antioquia department and a three-day course of artemether plus lumefantrine for the Cordoba department. As for gestational malaria, women in their 2^nd^ and 3^rd^ trimester of pregnancy were treated as non-pregnant subjects according to the place of residency, or with a 7- day course of quinine plus clindamycin if on their 1^st^ trimester of pregancy. In 2009, the scheme artesunate plus mefloquine was withdrawn as first-line from the national treatment guidelines and artemether plus lumefantrine is used throughout the country. Vivax malaria remains sensitive to a three-day course of chloroquine [17] and, in non-pregnant subjects, is administered in combination with a 14-day course of primaquine [18].

A study carried out in three municipalities of the Uraba region (north-west Colombia) recruited and followed >2,000 pregnant women at the antenatal clinics and hospitals to obtain a measure of incidenced from a group of 250 pregnant women recruited at delivery. Two studies explored the prevalence of gestational malaria and placental malaria at delivery in the Uraba region [8,9], yet no data are available from the Cordoba region, which, together with Uraba and Bajo Cauca, constitute the majority of malaria cases in Colombia [13,14].

The current study reports on the results of a study aimed at exploring the prevalence of gestational, placental and congenital malaria in women who delivered at the local hospitals of north-west Colombia, between June 2008 and April 2011. Diagnosis was based on the simultaneous application of two independent diagnostic tests: microscopy of thick blood smear and a PCR.

## Methods

### Study area

**Figure 1:**
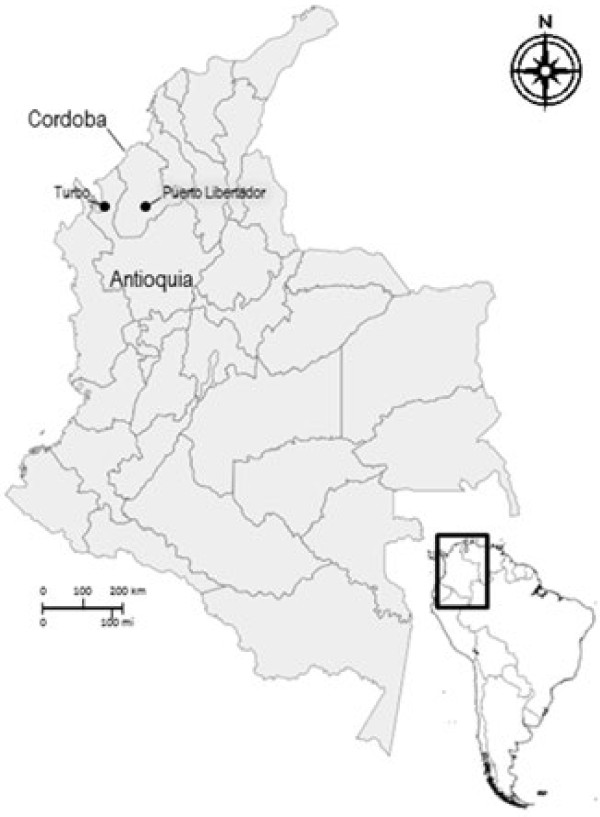
**Map of Colombia.** Turbo (Uraba region) of Antioquia department and Puerto Libertador (Cordoba) where the study took place, are located in north-west Colombia.

The study was carried out in north-west Colombia in the localities of Turbo (Antioquia department) and Puerto Libertador (Cordoba department) (Figure 1). Between Antioquia and Cordoba lays the malaria endemic region known as Uraba-Altos Sinu/San Jorge-Bajo Cauca, which has an estimated area of 43,506 km2 and a malaria at-risk population of 2.5 million distributed in 35 municipalities. The Antioquia department reported 20,511 (in 2008), 32,029 (in 2009) and 45,618 (in 2010) cases of malaria [19]. *Plasmodium vivax* was reported in 65–70% of total cases. Meanwhile, in Cordoba, during 2001–2009, in 1,433,461 inhabitants, a mean number of 37,616 cases/year were reported: 69% caused by *P. vivax*, 23% by *P. falciparum* and 1% from mixed infection. The municipalities of Valencia and Tierralta (south-west Cordoba, on the border with Uraba), and Puerto Libertador (south-east Cordoba) reported 90% of malaria cases in the region [20].

Subjects for the current study were recruited at the local hospitals “ESE Hospital Francisco Valderrama”, in Turbo, and “CAMU Divino Niño”, in Puerto Libertador. In these localities, the annual mean parasitic indexes (malaria cases/1000 inhabitants) during 2000–2009 were 46.6 in Turbo and 23.4 in Puerto Libertador [19,20].

### Study design and sample selection

The study was part of a larger project aimed at exploring the epidemiology (incidence and prevalence measures), clinical aspects (mother and child outcomes), and immunopathology (placental damage and immune regulation secondary to infection) of gestational and placental malaria in north-west Colombia, partial results have been reported on the frequency of infection and the histopathology of placental malaria [7,9,21]. A total 2,000 pregnant women were recruited for the main study at the localities of Turbo and Puerto Libertador. Subjects were recruited in a sequential fashion until the sample size for the main study was reached. Clinical and epidemiological surveys were applied to all those women. Data from medical records were also obtained in order to assess the clinical outcome of the pregnancy.

A subset of subjects was selected to explore the prevalence of the conditions in a descriptive, prospective and transversal (prevalence) design. A sample size of 129 pregnant women was calculated based on the following parameters: a total of 2,000 in-hospital deliveries during the 34 months of the study; 10% expected frequency of malaria (gestational malaria or placental malaria); 95% confidence interval; 5% sampling error. Subjects were selected and included in this study using a simple random sampling method from the records of the 2,000 women recruited in the main project. The prevalence of infection by *Plasmodium* was assessed intra-partum using two independent tests: microscopy of thick smears and PCR. For this, paired blood samples were obtained from peripheral vein of the mother and from the placenta at time of delivery.

### Inclusion and exclusion criteria

Inclusion criteria were > 1 year residency in the study region, general good health and no history of serious disease, signature of the voluntary consent form and hospital delivery. The only exclusion criterion was withdrawal of the voluntary consent.

### Term definitions

The following definitions were applied: A) Low birth weight: <2,500 grams. B) Premature neonate: <37 weeks. C) Intra-uterine growth restriction: ≥37 weeks of gestation and low birth weight. D) Abortion: delivery before 22 weeks of pregnancy. E) Stillbirth: dead neonate of >22 weeks. F) Anaemia: haemoglobin <11 g/dL. G) Full term pregnancy: >38 weeks. H) Gestational or pregnancy malaria: plasmodial infection diagnosed in a pregnant woman after examination of maternal peripheral blood. I) Placental malaria: plasmodial infection diagnosed in placenta after examination of placental blood.

### Sample preparation

Whole blood samples from mother´s peripheral blood and placenta were obtained at delivery rooms in the local hospitals of Puerto Libertador and Turbo. The mother`s sample was obtained by finger prick and placental blood was obtained from the lake formed following a wash with saline (0.9%) and removal of a 3x3x3 cm section in the area of the cord’s insertion on the maternal side of the organ. These samples were used to perform thick and thin smear examination and PCR as detailed below.

### Diagnosis of *Plasmodium* infection

The presence of *Plasmodium* was confirmed in maternal, placental and cord blood by thick smear and a nested PCR according to standard published methods [9,22,23]. Diagnosis by microscopy was carried out in the field site by a trained technician with certified experience in malaria diagnosis. Parasitaemia was calculated after observation of fields corresponding to 200 leukocytes and a constant of 8,000 leucocytes/μL. A sample was considered negative after observation of 500 high power fields.

Diagnosis of infection by PCR was performed to whole blood samples collected onto Whatman 3MM filter paper, from which DNA was extracted using Chelex100® (Sigma™). Amplification was carried out using a nested PCR assay to detect the 18 s rRNA gene of *P. falciparum* and *P. vivax*, according to previously published procedures [23]. Positive reaction controls consisted of DNA from the laboratory-adapted strain *P. falciparum* 3D7 and from a well characterized field isolate of *P. vivax*. Amplification products were resolved in a 2% agarose gel using ethidium bromide and visualized under UV light. Samples were processed once as long as controls were confirmed to operate in optimal conditions, otherwise, the assay was repeated until successful performance of controls.

### Statistical analyses

The prevalence of gestational malaria and placental malaria was calculated according to the results of thick smear microscopy and PCR. The association between the distribution of frequencies among two variables was identified using χ^2^. Significance was established at p < 0.05%. Data analyses were performed using EpiInfo® 6.0 and SPSS® 10.0.

### Ethical aspects

The study protocol was reviewed and approved by: a) Ethics Committee of the Sede de Investigaciones Universitarias SIU, Universidad de Antioquia (Medellín, Colombia) (acta 07-32-126: project Colciencias code 111540820495, contract: 238–2007); b) Ethics Committee of the Instituto de Investigaciones Medicas, Universidad de Antioquia (acta 012: project Colciencias code 111549326134, contract 611 de 2009); c) Ethics Committee of Instituto de Investigaciones Medicas, Universidad de Antioquia (Medellin, Colombia) (project Regionalización-Universidad de Antioquia IIM 8764–2530). Each participant gave full informed consent according to the Helsinki convention and the Colombian regulations for this type of research. Each subject voluntarily accepted to participate in the study.

Women with a positive peripheral blood thick smear were treated, before hospital discharge, according to the recommendation of the Ministry of Health: a three-day course of chloroquine (25 mg/kg total dose) plus a 14-day course of primaquine (0.25 mg/kg/day) in *P. vivax* infection, or, in *P. falciparum*, a 3-day course of artesunate plus mefloquine at 200 mg and 500 mg, respectively, per day in subjects aged >13 years (Antioquia department) or artemether plus lumefantrine (Coartem®) twice a day for a total six doses in subjects >34 kg (Cordoba department) [18].

## Results

During the 34 months of the study (June 2008 - April 2011), a total of 121 parturient women were recruited at the local hospitals (eight records, out of the 129 expected subjects, had incomplete information). In general, subjects were young (mean age = 21.9 ± 6.7), the mean number of gravidity, including the current pregnancy, was 3.2 ± 2.9; all (but one) had a full term pregnancy (38.8 ± 2.1 weeks), and their mean haemoglobin level was 10.6 ± 0.13 g/dL (mean 10.3 g/dL in thick smear-gestational malaria positive and mean10.6 g/dL in thick smear-gestational malaria negative, p >0.05). Review of clinical records confirmed that attendance to the antenatal clinic was late, scarce and irregular with a mean number of clinical evaluations of three and < 20% of mothers receiving > 5 evaluations. In addition, application of a VDRL test during the antenatal period was performed in 70 subjects and one woman tested positive (titer 1:8). A total of 48 subjects were tested for HIV and all were negative. All participants were asymptomatic for malaria infection at delivery, and insecticide-treated nets were not used.

The frequency of gestational malaria was 9.1% (11/121) by microscopy, and 14.0% (17/121) by PCR (Table 1). Meanwhile, the frequency of placental malaria was 3.3% (4/121) by microscopy and 16.5% (20/121) by PCR. In cases of gestational malaria, the frequency of submicroscopic parasitaemia (*i.e*. a positive PCR with negative microscopy) was 4.9% (14.0–9.1), and in cases of placental malaria this was 13.2% (16.5–3.3). The ratio between positive microscopy and positive PCR in gestational malaria was 1:1.5, and in placental malaria this was 1:5.

**Table 1:** Comparison of frequencies of gestational and placental malaria according to thick smear microscopy and PCR

**A. Gestational Malaria**				
	**PCR**			
	**Negative**	***P. vivax***	***P. falciparum***	**Total**
**Microscopy**				
**Negative**	103	4	3	110
***P vivax***	0	7	0	7
***P falciparum***	1	0	2	3
**Mixed**	0	0	1	1
**Total**	104	11	6	121
**B. Placental Malaria**				
	**PCR**			
	**Negative**	***P. vivax***	***P. falciparum***	**Total**
**Microscopy**				
**Negative**	101	5	11	117
***P vivax***	0	2	0	2
***P falciparum***	0	0	2	2
**Total**	101	7	13	121

Among the 17 cases of gestational malaria diagnosed by PCR, 65% (11/17) were *P. vivax*, and 35% (6/17) were *P. falciparum.* The opposite was observed in placental malaria: *P. falciparum* was detected in 65% (13/20), and *P. vivax* in 35% (7/20) of cases. All samples of umbilical cord blood were negative for malarial parasites by both tests.

Concurrence of gestational and placental malaria by microscopy was absent in the 110 subjects negative by microscopy and 36% of the 11 subjects with gestational malaria evidenced placental infection. The frequency of simultaneous maternal and placental infection increased when PCR was performed: in the 104 gestational malaria negative subjects, 92% (96/104) were placental malaria free, and in the 17 gestational malaria positive subjects, 71% (12/17) exhibited placental malaria. Therefore, the presence of parasites in the peripheral mother`s blood was not always associated with placental infection, but absence of parasites in the mother`s blood did not necessarily imply that the placenta was free of infection (Table 2).

**Table 2:** Correlation between diagnosis of gestational malaria and placental malaria by two diagnostic methods (A. Thick smear microscopy and B. PCR)

**A. Thick smear microscopy**				
	**Placental malaria**			
	**Negative**	***P. vivax***	***P. falciparum***	**Total**
**Gestational malaria**				
**Negative**	110	0	0	110
***P vivax***	5	2	0	7
***P falciparum***	2	0	1	3
**Mixed**	0	0	1	1
**Total**	117	2	2	121
**B. PCR**				
	**Placental malaria**			
	**Negative**	***P. vivax***	***P. falciparum***	**Total**
**Gestational malaria**				
**Negative**	96	1	7	104
***P vivax***	5	6	0	11
***P falciparum***	0	0	6	6
**Total**	101	7	13	121

History of gestational malaria was confirmed by review of clinical records in 26% (31/120); cases were diagnosed by microscopy of mother´s peripheral blood and were treated according to the Ministry of Health guidelines for malaria in pregnancy by the time of diagnosis and as detailed in the introduction section. In 87% (27/31) of cases of gestational malaria before delivery, at least one of the following was recorded: *Plasmodium* species, parasitaemia, or the gestational age at the time of diagnosis. When species was recorded (18/27 or 67%), 78% corresponded to *P. vivax*, and the remaining were *P. falciparum.* The mean parasitaemia in *P. falciparum* infections was 2,771 ± 4,876 parasites/μL (median: 617; range: 40--18.060).

Intrapartum microscopy or PCR of peripheral blood confirmed infection in up to 35.5% (11/31) of the subjects with a history of gestational malaria (73% *P. vivax*, 18% *P. falciparum,* 9% mixed). In the 89 mothers without a history of gestational malaria, infection was detected at delivery in 7% (3.5% each *P. vivax* and *P. falciparum*). A report of gestational malaria in the current pregnancy was significantly associated (p = 0.000121) with a positive PCR test at delivery and 94% (84/89) of the women without a history of malaria were negative for gestational malaria at delivery (Table 3).

**Table 3:** Correlation of diagnosis of gestational or placental malaria at delivery by PCR and a history of gestational malaria during pregnancy

A. Gestational malaria*				
	**Negative**	***P. vivax***	***P. falciparum***	**Total**
**History of gestational**				
**malaria**				
**Negative**	84	3	2	89
**Positive**	20	8	3	31
**Total**	104	11	5	120
**B. Placental malaria**^**+**^				
	**Negative**	***P. vivax***	***P. falciparum***	**Total**
**History of gestational**				
**malaria**				
**Negative**	83	1	5	89
**Positive**	18	6	7	31
**Total**	101	7	12	120

*p = 0.000121 for species, p = 0.000094 for presence-absence of infection, ^+^p = 0.000010 for species and p = 0.000014 for presence-absence of infection.

According the place of residency, prevalence of gestational malaria was 4% (3/71) in Turbo and 28% in Puerto Libertador (14/50). Meanwhile, the prevalence of placental malaria was 4% (3/71) and 34% (17/50), respectively. Therefore, both gestational and placental malaria were significantly associated to the place of residency (p = 0.001040 and p = 0.000049, respectively) (Table 4). No significant association could be confirmed between parity and presence of malarial infection by any of the tests applied (p > 0.153), which means that regardless of being in their first, second, third or >3rd pregnancy, all women had the same risk of gestational malaria and placental malaria.

**Table 4:** Frequencies of diagnosis of gestational and placental malaria at delivery by PCR according to locality

A. Gestational malaria*				
	**Negative**	***P. vivax***	***P. falciparum***	**Total**
**Turbo**	68	2	1	71
**Puerto Libertador**	36	9	5	50
**Total**	104	11	6	121
**B. Placental malaria**^**+**^				
	**Negative**	***P. vivax***	***P. falciparum***	**Total**
**Turbo**	68	0	3	71
**Puerto Libertador**	33	7	10	50
**Total**	101	7	13	121

*p = 0.001040 for species, p = 0.000211 for presence-absence of infection, ^+^p = 0.000049 for species and p = 0.000014 for presence-absence of infection.

In one subject a stillbirth was reported. This occurred in an 18 year-old mother in her first pregnancy and with a history of gestational malaria by *P. vivax*. Maternal haemoglobin at delivery was 9.1 g/dL and the gestational age was 27 weeks. Malaria-specific results for this case confirmed maternal infection with *P. vivax* and *P. falciparum* by microscopy whereas only *P. falciparum* was detected by PCR. Placental infection by *P. falciparum* was detected by both tests and the cord blood was negative for infection.

## Discussion

The current report on the prevalence of gestational, placental and congenital malaria in parturient women from a highly malaria endemic region of Colombia, further defines the epidemiology of this infection in the country. A major limitation of the study is the absence of follow-up at the antenatal clinic and the recruitment in a passive fashion. This resulted in the inclusion of a population which might not precisely reflect the dimension of the malaria problem during pregnancy. Nevertheless, the fact that all studied subjects were asymptomatic for malaria, suggests that the figures herein published underestimate the actual frequency of gestational malaria. Current ongoing studies, overcome this limitation by including recruitment at the antenatal clinics and follow-up until delivery.

It is important to highlight that during the recruitment of subjects for the current study, the health authorities of Uraba carried out an intense malaria control programme from 2007 until 2009 with a major impact on the frequency of malaria, as reported herein. The programme, named “Papa Luis”, consisted of massive anti-malarial administration (chloroquine), biologic control of *Anopheles spp.* larvae, swamp drainage and indoor residual spraying. A programme as such was not implemented in Cordoba. In addition, both study regions were deeply affected by intense and continuous rainy seasons during 2008, 2010 and the first six months of 2011, which also contributed to a reduction in the reported number of cases concomitantly with the rains, followed by a rise of cases as they decline. Finally, attendance of mothers at any health facility was seriously affected by the political instability and social insecurity prevalent in the region. All these factors contributed to reduced attendance at hospitals, increased the recruitment period and, very probably, impacted the results after inclusion of subjects resident in and around urban centers. Nevertheless, in Puerto Libertador the frequency of gestational malaria was seven times that recorded in Turbo (4% *vs*. 28%) and placental malaria was eight-fold that observed in Turbo (4% *vs.* 34%). Climate conditions and turmoil from violence are similar in both regions and only the application of control programmes seems to differ between the two localities. Major fluctuations in the frequency of gestational and pregnant malaria have also been reported in other endemic countries [24,25].

Studies on gestational placental or congenital malaria are scarce in America in comparison to Africa [4-6,26,27] and most recent reports come from Colombia [7,9-12] and Brazil [4,26]. In the Brazilian Amazon, among 1,699 women with malaria, the prevalence of gestational malaria was 11.7% and *P. vivax* was confirmed in 83% of cases [25]. In a previous report from Rio Branco (Brazil) among 33,420 women who attended the hospital, 445 had gestational malaria resulting in a prevalence of 1.4%; *P. vivax* was detected in 53% of them [24]. In the current report, the prevalence of gestational malaria was 9.1% by microscopy and 14% by PCR, while the prevalence of placental malaria was 3.3% by microscopy and 16.5% by PCR. The positivity rates by microscopy, for the both gestational and placental malaria, are lower than those previously reported in 2004–2006 in the same region (10-14% gestational malaria and 9-12% placental malaria) [7-9], probably as a result of the intervention measures and the climate conditions above detailed.

As for congenital malaria, the current report confirmed previous observations made by other authors on the low frequency of infection in cord blood (2.5%) in relation with higher rates of gestational malaria (9.5%) [11]. Several factors might explain the relative protection of the foetus to acquisition of congenital malaria, namely the degree of previously acquired immunity of the mother [28], the proportion of foetal haemoglobin [28], and the higher frequency of *P. vivax* infection, which in theory, fails to sequester in placenta, among others. The fact that subjects recruited in this study were asymptomatic yet they presented with parasitaemia, strengthens the important role of passive transfer of maternal anti-malarial antibodies in the protection against congenital malaria. This subject is under exploration in specifically-designed ongoing studies.

Attendance to the antenatal clinic was inadequate in coincidence with previous reports from the region [7], and the number of women tested for infections such as syphilis and HIV was alarmingly low. Insecticide-treated net usage is uncommon in Colombia (< 20%), as is indoor residual spraying [29]. A similar situation is seen in other countries of the Americas. The complete failure to use insecticide-treated nets by subjects enrolled in this study, reflects the lack of government will to effectively address the malaria problem. Furthermore, intermittent preventive therapy during pregnancy is not applied on a routine basis in Colombia or elsewhere in the Americas, despite being a proven control measure in most African countries [30]. Failure to apply any of the above-mentioned control measures, in addition to the lack of access to adequate antenatal care, the precarious life conditions and the extreme political violence of most malaria endemic regions perpetuate the high risk of malaria transmission, among other infectious conditions, in mothers and their children.

The results reported herein confirm the higher frequency of gestational and placental malaria detected by PCR compared to microscopy as reported previously in this and other endemic regions [9,31,32]. Furthermore, recent studies in Colombia confirm the higher sensitivity of a quantitative PCR over a standard nested PCR, to detect gestational and placental malaria [33]. However, in field conditions, application of any molecular based approach remains impractical and innovative solutions are highly welcomed by field researchers. Molecular tools allow identification of sub-microscopic infections, which in the current studied represented 4.9% of gestational malaria and 13.2% of placental malaria, but no effect on newborn`s weight or mother’s haemoglobin could be confirmed. A recent review on submicroscopic *Plasmodium* infection during pregnancy concluded that the risk of anaemia and low birth weight infants are increased in positive cases [34]. Following up studies, with precise assessment of clinical outcomes in infected cases might help to elucidate the consequences of submicroscopic malaria during pregnancy in this region of the continent.

The presence of a positive peripheral thick smear at delivery seems insufficient to predict the existence of placental infection, but a negative smear cannot guarantee an uninfected placenta. Therefore, due to the potential risk of malaria in the mother, clinicians should presume placental infection in all cases of gestational malaria. Of major importance in the clinical practice is the finding that a positive history of gestational malaria correlated with gestational and/or placental malaria at delivery, regardless of adequate anti-malarial treatment of the original episode. Since *P. falciparum* seems to remain susceptible to artemisinin-based combination therapy [35], possible explanations for placental parasite persistence include sequestration of parasites in the placenta, *P. vivax* relapses and erroneous species identification (a documented limitation of microscopy) during the primary episode.

The current report confirms the findings of previous researchers which report gestational and placental malaria as serious public health problems in north-west Colombia. Moreover, during the period of study, the local health authorities failed to observe the official recommendation since routine thick smear of peripheral blood was not practiced at antenatal visits, delaying diagnosis and treatment. This, combined with the social inequity faced by the women and families inhabiting the region; and the particular cultural frame and living conditions, contributes to perpetuate the problem [36,37]. Malaria in Colombia is a disease of deprived and economically unstable populations, which are also poorly attended by the health services. Among them, women are the most vulnerable from the social perspective since they are particularly susceptible to develop malaria complications during pregnancy and lack access to economic and health resources [38-40].

## Conclusions

The following conclusions were reached: 1) the problem of malaria during pregnancy and placental malaria is serious in north-west Colombia; 2) the history of gestational malaria constitutes an indicator of gestational and placental malaria at delivery; 3) the risk of congenital malaria is low; 4) measures should be implemented by regional and national health authorities to guarantee that recommendations regarding malaria testing at the antenatal clinics are fulfilled.

## Competing interests

The authors declare that they have no competing interests.

## Authors’ contributions

OA and EA performed field work and processed blood samples. AM supervised overall design and development and wrote the manuscript, JC conceived the Project, designed the protocol and supervised overall design and development. All authors read and approved the final manuscript.
